# Chloroplast Genome Insights of *Gleditsia japonica* var. *velutina* and Evolutionary Implications for Variation and Phylogeny in *Gleditsia*

**DOI:** 10.1002/ece3.73826

**Published:** 2026-06-11

**Authors:** Shiyu Hu, Naiwei Li, Xingjian Liu, Baocheng Wu, Yueqi Sun, Mimi Li

**Affiliations:** ^1^ Institute of Botany Jiangsu Province and Chinese Academy of Sciences Nanjing China; ^2^ Jiangsu Key Laboratory for Conservation and Utilization of Plant Resources Nanjing China

**Keywords:** codon usage bias, *Gleditsia japonica* var. *velutina*, mechanism of expansion, molecular markers, phylogenetic analysis

## Abstract

The genus *Gleditsia* possesses significant medicinal value, yet the Chinese endemic *G. japonica* var. *velutina* is threatened. Here, we integrated Nanopore long‐read and Illumina short‐read sequencing to assemble the complete chloroplast genome of *G. japonica* var. *velutina*, and perform a systematic analysis of its evolutionary characteristics. The results demonstrate that chloroplast genome size variation within the genus (162.4–170.8 kb) is primarily driven by expansions of repeat sequences in the large single‐copy (LSC) region. Genome‐wide comparisons revealed hypervariable intergenic regions and polymorphic genes as potential novel molecular markers for species delimitation within *Gleditsia*, particularly for the endangered *G. japonica* var. *velutina*. Comparative analysis further showed that codon usage bias is predominantly shaped by natural selection and remains highly conserved across the genus, with 30 optimal codons identified. Phylogenomic analyses support the close relationship of *G. japonica* var. *velutina* with *G. japonica* var. *delavayi* and *G. japonica* . This study presents the first comprehensive chloroplast genome analysis of the critically endangered *G. japonica* var. *velutina*, providing essential insights into its evolutionary divergence within the genus *Gleditsia* and offering a robust genomic foundation for its conservation.

## Graphical abstract

This study presents the first comprehensive chloroplast genome analysis of the critically endangered *G. japonica* var. *velutina*, providing essential insights into its evolutionary divergence within the genus *Gleditsia* and offering a critical genomic foundation for its conservation.

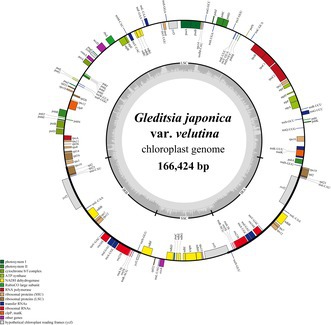

## 1 Introduction

The genus *Gleditsia* (Fabaceae: Caesalpinioideae) is primarily distributed across the temperate and subtropical regions of Central Asia, Southeast Asia, and the Americas (Huang 2015; Li 2007). Eight species occur in China, including *G. japonica* , *G. japonica* var. *delavayi*, and *G. japonica* var. *velutina* (Lan et al. 2004). *Gleditsia* species possess significant medicinal value and are rich in flavonoids, indole alkaloids, galactomannan, and triterpenoid saponins, leading to their widespread use in traditional Chinese medicine for treating headache, expectoration, and asthma (Zhang et al. 2016; Ashraf et al. 2022). Modern pharmacology has confirmed its potential as a phytomedicinal source, with compounds exhibiting inhibitory effects against hyperglycemia, hyperlipidemia (Liu et al. 2024; Sun et al. 2018), and HIV replication (Chang et al. 2024; Vlietinck et al. 1998).

*G. japonica* var. *velutina* faces an imminent extinction crisis. Fewer than 10 wild individuals remain, restricted to a narrow elevational range below 950 m in stream valleys of Mount Heng, Hunan Province. Accordingly, it is designated as a Class I National Key Protected Wild Plant of China (Su and Xv 2023; Qin et al. 2023) and classified as Critically Endangered by the International Union for Conservation of Nature (IUCN) (World Conservation Monitoring Centre 1998). Successful reproduction in *Gleditsia* depends critically on synchronized flowering and pollen transfer from male plants. However, *G. japonica* var. *velutina* exhibits asynchronous maturation of female and male flowers, resulting in severely impaired natural pollination efficacy (Liu et al. 2023). Dichogamy further exacerbates its vulnerability under climate change, increasing risks of phenological mismatches, pollinator scarcity, and skewed sex ratios, imposing substantial reproductive stress. Additionally, the densely velutinous, leathery pods are curved, with poor dehiscence upon maturity. while the hard seed coat impedes water imbibition, resulting in low natural germination rates and thus compromised reproductive capacity (Ahmed 2015).

Effective conservation management for endangered plants relies on a robust understanding of their genetic and evolutionary context (Escudero et al. 2003). The chloroplast (cp) genome, characterized by low recombination rates and slow evolutionary rates, is pivotal for precise species identification and inferring deep evolutionary history (Dong et al. 2021, 2022), making it essential for assessing genetic differentiation and diversity in the genomic era. Furthermore, its maternal inheritance pattern renders the cp genome an excellent tool for tracking historical gene flow directions and hybridization events (Huang et al. 2014; Perdereau et al. 2017). This enables the detection and assessment of hybridization threats to the genetic integrity of endangered species, providing a scientific basis for implementing isolation measures or removing hybrid individuals.

Despite previous studies on *Gleditsia* cp genomes (Xiao et al. 2024; Tan et al. 2020), a comprehensive investigation of the critically endangered *G. japonica* var. *velutina* at the cp genome level has been conspicuously absent. Consequently, the patterns of sequence divergence and evolutionary connections to its relatives remain unclear. While Illumina short‐read sequencing has enabled cp genome assembly, it often struggles to accurately resolve long repeat regions, a common feature of chloroplast DNA. To overcome this limitation, this study employed a hybrid sequencing approach using both Illumina short‐read and Nanopore long‐read technologies to construct the complete chloroplast genome of *G. japonica* var. *velutina* and conduct a comparative analysis with closely related species. By comparing the complete chloroplast genomes of representative *Gleditsia* species, this study aims to elucidate evolutionary relationships in the genus and evaluate the taxonomic status of *G. japonica* var. *velutina*. In addition, considering its endangered status, we aim to provide a genetic basis for its reliable identification and effective conservation.

## 2 Materials and Methods

### 2.1 Genomic DNA Extraction and Sequencing

Fresh leaves were collected from a single cultivated sapling of *G. japonica* var. *velutina* at the Nanjing Botanical Garden Mem. Sun Yat‐Sen. A voucher specimen has been deposited in the Herbarium of Institute of Botany, Jiangsu Province and Chinese Academy of Sciences (NAS) under accession number NAS00818519. Total genomic DNA was isolated using the CTAB method (Doyle and Doyle 1987). The extracted DNA underwent stringent quality evaluation: purity was assessed using a NanoDrop spectrophotometer and concentration was accurately quantified using the Qubit 3.0 Fluorometer, while integrity was checked using agarose gel electrophoresis.

For short‐read sequencing, paired‐end libraries with insert sizes of 350‐bp were constructed and sequenced on the Illumina NovaSeq 6000 platform (Illumina, CA, USA) to attain ≥ 50× coverage (Batzoglou et al. 1999). For long‐read sequencing, DNA fragments exceeding 20 kb were size‐selected using a BluePippin system (Sage Science, MA, USA). A sequencing library was prepared using the SQK‐LSK109 kit (Oxford Nanopore Technologies, Oxford, UK). Libraries were measured using a Qubit fluorometer and subsequently processed on a PromethION R10.4.1 flow cell (Oxford Nanopore Technologies, Oxford, UK) to acquire lengthy read length data encompassing intricate repetition areas.

### 2.2 Chloroplast Genome Assembling and Annotation

PtGAUL v1.05 (Zhou et al. 2023) was used for preliminary assembly of the Nanopore long‐read data, followed by error correction and polishing with Illumina short reads using Pilon v1.24 (Walker et al. 2014). The genome was annotated using the online platforms CPGavas2 (http://47.96.249.172:16019/analyzer/annotate) (Shi et al. 2019) and further manually refined with GeSeq (https://chlorobox.mpimp‐golm.mpg.de/geseq.html) (Tillich et al. 2017) and Geneious Prime v2025.02 (Kearse et al. 2012), incorporating comparisons with reference cp genomes. The complete chloroplast genome map was visualized using the Organellar Genome DRAW (OGDRAW) tool (https://chlorobox.mpimp‐golm.mpg.de/OGDraw.html) (Greiner et al. 2019). The final annotated chloroplast genome of *G. japonica* var. *velutina* was compared with those of other *Gleditsia* species retrieved from NCBI, including *G. japonica* (NC_047281), *G. japonica* var. *delavayi* (NC_070139), *G. fera* (NC_070139), *G. microphylla* (OP722581), and *G. sinensis* (NC_047282) to elucidate the genetic variations within the genus.

### 2.3 Codon Usage Analysis

CodonW v1.4.4 (Sun et al. 2013) and EMBOSS (https://www.bioinformatics.nl/emboss‐explorer/) (Rice et al. 2000) were used to calculate GC content at the third codon position (GC3s), effective number of codon (ENCs), and relative synonymous codon usage (RSCU) (Dorić and Bilela 2014; Blake et al. 2003; Sharp et al. 1986). The synonymous codon usage order (SCUO) was calculated using CU.Win2000 v2.0 (Wan et al. 2006) to quantify the degree of codon usage bias, with higher SCUO value indicating stronger bias. The R package coRdon v1.13 (https://bioconductor.org/packages/devel/bioc/vignettes/coRdon/) was used to compute gene expression levels based on measures independent of length and composition (MILC).

RSCU values exceeding 1 indicate positive preference, while values below 1 indicate a negative preference. Codons with RSCU > 1 and ΔRSCU > 0.08 are classified as optimal codons (Xiao, Hu, et al. 2024). The ENC‐plot (ENC vs. GC3s) was plotted to analyze the factors affecting codon usage patterns (Wu et al. 2018). The points falling on or near the expected curve suggest a dominant role of mutation pressure, whereas points falling below the curve indicate the influence of natural selection (Wright 1990). The relative roles of mutation and selection were further evaluated using a PR2‐plot (Xiao, Hu, et al. 2024; Sueoka 1999a) and neutrality plot (Sueoka 1999b; He et al. 2016). The PR2 plot was used to evaluate whether the base composition at the third codon position deviated from parity. Specifically, A3/(A3 + T3) and G3/(G3 + C3) values close to 0.5 indicate balanced usage of complementary bases, whereas deviations from 0.5 suggest the influence of natural selection. The neutrality plot, based on the regression of GC12 against GC3, was used to infer the relative contributions of mutational pressure and natural selection to codon usage bias. A regression slope close to 1 indicates a predominant role of mutational pressure, while a slope close to 0 indicates stronger selective constraints.

### 2.4 Repeat Elements Analysis

Tandem repeat sequences were identified using Tandem Repeats Finder (https://tandem.bu.edu/trf/trf.html) (Benson 1999), with alignment parameters settings set to 2, 7, and 7 for match, mismatch, and indel, respectively. The minimum alignment score and maximum cycle size were 50 and 500, respectively. Repeat sequence analysis of the chloroplast genome was conducted using REPuter (https://wwww.cebitec.uni‐bielefeld.de/) (Kurtz et al. 2001). Four types of repeats were detected, including forward, reverse, palindromic, and complementary repeats. The parameters were set to a minimum repeat length of 30 bp and a minimum sequence identity of 90% between repeat copies. Simple sequence repeats (SSRs) were identified using MISA (https://webblast.ipk‐gatersleben.de/misa/) (Beier et al. 2017). For SSR detection, the minimum repeat number thresholds were set to 10, 6, 5, 4, 3, and 3 repeat units for mono‐, di‐, tri‐, tetra‐, penta‐, and hexanucleotide motifs, respectively. All identified duplicates were manually validated, and the redundant segments were removed.

### 2.5 Sequence Divergence Analysis

Comparative analyses of interspecific differences were performed using the mVISTA (https://genome.lbl.gov/vista/mvista/) (Frazer et al. 2004). Variant sites were identified with Geneious Prime v2025.02 (Kearse et al. 2012), and chloroplast genome boundary differences were examined using IRscope (https://irscope.shinyapps.io/irapp/) (Amiryousefi et al. 2018). Whole chloroplast genomes of six *Gleditsia* species were aligned using MAFFT v7.526 (Katoh and Standley 2013), and sequence polymorphism was analyzed by DnaSP v6.0 (Rozas et al. 2017), with a window length of 2000 bp and a step size of 25 bp. Coding sequences from *Gleditsia* species were retrieved, and non‐synonymous (Ka) and synonymous (Ks) substitution rates were calculated using TBtools v2.475 (Chen et al. 2023).

### 2.6 Phylogenetic Analysis

To investigate the evolutionary relationships within the subfamily Caesalpinioideae, we conducted a phylogenetic analysis based on chloroplast genomes. The ingroup dataset, focused exclusively on Caesalpinioideae, comprised 40 accessions representing five major clades. The *Umtiza* clade comprised the genus *Gleditsia* along with two *Gymnocladus* species: *Gy. chinensis* (OP722582) and *Gy. dioicus* (NC_070141). *Glycine max* (Papilionoideae, NC_007942) was included as the outgroup taxon to root the phylogenetic tree. Sequences of coding and non‐coding regions were extracted by Phylosuite v2.0 (Zhang et al. 2020), multiple sequence alignment was conducted with MAFFT v7.526 (Katoh and Standley 2013), and the results were refined with TrimAL v1.5.0 (Capella et al. 2009). The best‐fit nucleotide substitution model was identified using JModelTest v2.0 (Darriba et al. 2012) based on the Akaike information criterion (AIC), with the GTR + I + G model selected as the most suitable for subsequent phylogenetic analysis. Phylogenetic trees were constructed using Maximum Likelihood (ML) with PhyML v3.0 (Guindon et al. 2010), employing 1000 bootstrap replicates to evaluate support. For Bayesian inference, MrBayes v3.2 (Ronquist et al. 2012), which conducted Markov Chain Monte Carlo (MCMC) analysis with two independent chains for 10,000,000 generations each, sampling every 1000 generations. The initial 25% of samples are discarded as burn‐in, and the remaining samples are utilized to formulate a consensus tree based on a 50% majority rule. Convergence was considered reached when the average standard deviation of split frequencies falls below 0.01, and the final phylogenetic tree is produced.

## 3 Results

### 3.1 Complete Chloroplast Genome of *G. japonica* Var. *velutina*

**FIGURE 1:**
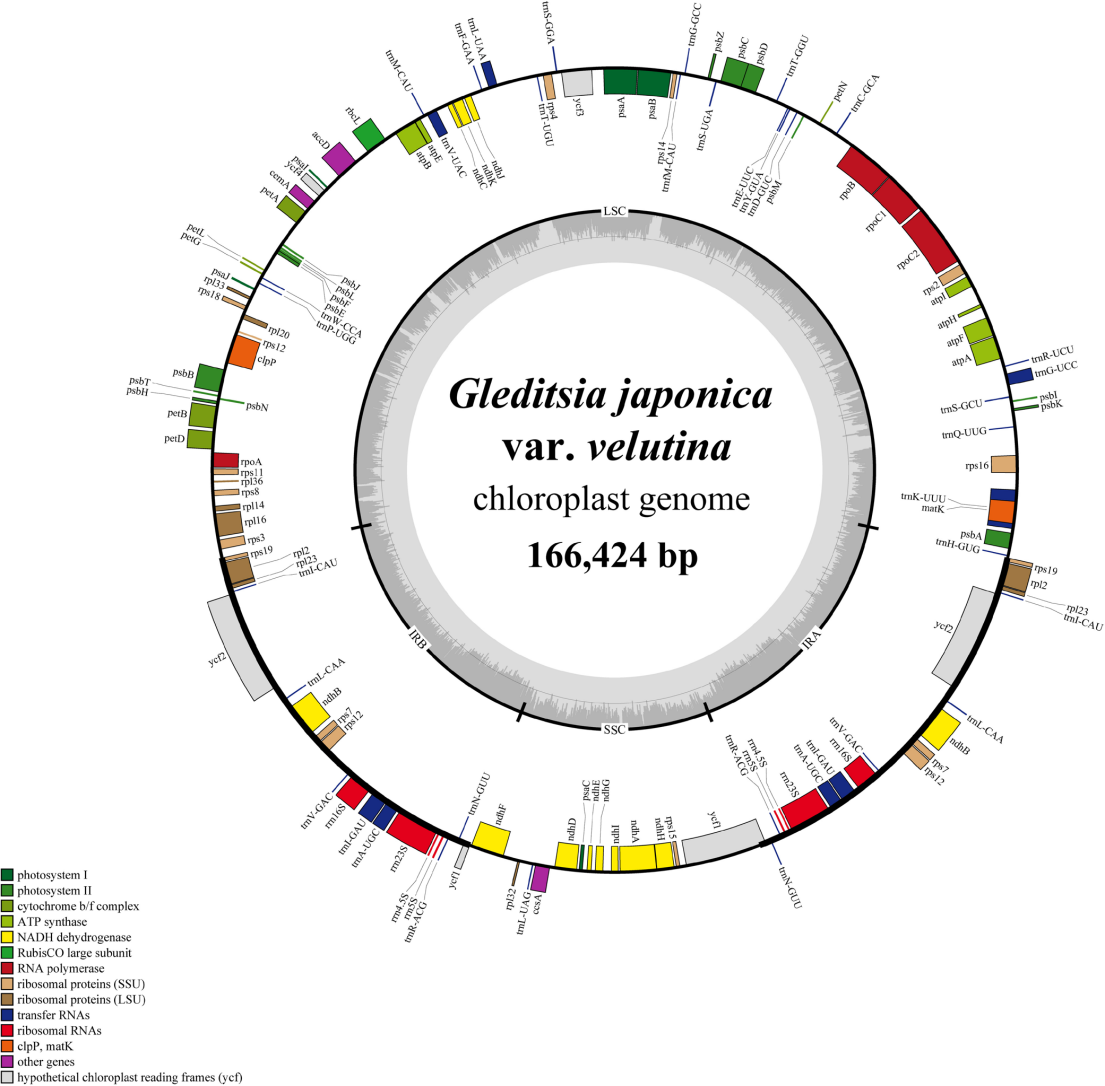
Chloroplast genome map of *Gleditsia japonica* var. *velutina*.

The chloroplast (cp) genome of *G. japonica* var. *velutina* was assembled, resulting in a 166,424 bp circular genome (Figure 1) comprising a large single‐copy region (LSC) of 94,841 bp, a small single‐copy region (SSC) of 19,503 bp, and two inverted‐repeat regions (IRs) of 26,040 bp, exhibiting a typical quadripartite structure. The overall GC content was 34.8%, and the genome contained 111 unique genes, including 77 protein‐coding genes, 30 tRNA genes, and 4 rRNA genes (Table 1). Two protein‐coding genes (*clpP* and *ycf3*) each contained two introns (Table 2), whereas ten other protein‐coding genes contained a single intron (*rps12*, *rps16*, *atpF*, *rpoC1*, *petB*, *petD*, *rpl2*, *rpl16*, *ndhB*, and *ndhA*). The IR region comprised 19 duplicated genes, including four rRNA genes (*rrn4.5*, *rrn5*, *rrn16*, *rrn23*), seven tRNA genes (*trnA‐UGC*, *trnL‐CAA*, *trnI‐GAU*, *trnI‐CAU*, *trnN‐GUU*, *trnR‐ACG*, *trnV‐GAC*), and eight protein‐coding genes (*rpl2*, *rpl23*, *rps7*, *rps12*, *rps19*, *ndhB*, *ycf1*, *and ycf2*). Notably, *rps19* and *ycf1* were not fully duplicated in the IR regions. In addition, *rps12* exhibits a typical trans‐splicing structure, with exon 1 located in the LSC region and exons 2 and 3 located in the IR regions (Figure 1).

**TABLE 1:** Comparison of chloroplast genome among *Gleditsia* species. Values in parentheses indicate duplicated gene copies located in the IR regions.

Characteristics	*G. japonica* var. *velutina*	*G. japonica* var. *delavayi*	*G. japonica*	*G. fera*	*G. microphylla*	*G. sinensis*
Size (bp)	166,424	170,796	162,391	165,027	170,713	163,175
LSC (bp)	94,841	98,889	90,870	93,345	98,595	91,540
SSC (bp)	19,503	19,561	19,449	19,374	18,880	19,250
IR (bp)	26,040	26,173	26,036	26,154	26,619	26,193
Protein‐coding regions (bp)	78,705	78,609	78,747	78,603	78,876	78,162
Number of total genes	130	130	130	130	130	130
Number of protein‐coding genes	85 (8)	85 (8)	85 (8)	85 (8)	85 (8)	85 (8)
Number of tRNA genes	37 (7)	37 (7)	37 (7)	37 (7)	37 (7)	37 (7)
Number of rRNA genes	8 (4)	8 (4)	8 (4)	8 (4)	8 (4)	8 (4)
Overall GC content (%)	34.80	33.90	35.50	35.20	34.00	35.60
GC content in LSC (%)	31.60	30.30	32.70	32.20	30.40	32.80
GC content in SSC (%)	29.30	29.30	29.30	29.40	29.40	29.50
GC content in IR (%)	42.60	42.50	42.70	42.50	42.20	42.50

**TABLE 2:** Composition of the chloroplast genome of *Gleditsia japonica* var. *velutina*.

Groups of genes	Name of genes
Ribosomal RNAs	*rrn4.5*, *rrn5*, *rrn16*, *rrn23*
Transfer RNAs	*trnA‐UGC* ^a^, ^b^, *trnC‐GCA*, *trnD‐GUC*, *trnE‐UUC*, *trnF‐GAA*, *trnG‐UCC* ^a^, *trnG‐GCC*, *trnH‐GUG*, *trnI‐GAU* ^a^, ^b^, *trnK‐UUU* ^a^, *trnL‐CAA*, *trnL‐UAA* ^a^, *trnL‐UAG*, *trnfM‐CAU*, *trnI‐CAU*, *trnM‐CAU*, *trnN‐GUU*, *trnP‐UGG*, *trnQ‐UUG*, *trnR‐ACG*, *trnR‐UCU*, *trnS‐GCU*, *trnS‐GGA*, *trnS‐UGA*, *trnT‐GGU*, *trnT‐UGU*, *trnV‐GAC*, *trnV‐UAC* ^a^, *trnW‐CCA*, *trnY‐GUA*
Large units of ribosome	*rpl2* ^a^, *rpl14*, *rpl16* ^a^, *rpl20*, *rpl23*, *rpl32*, *rpl33*, *rpl36*
Small units of ribosome	*rps2*, *rps3*, *rps4*, *rps7*, *rps8*, *rps11*, *rps12* ^a^, ^b^, *rps14*, *rps15*, *rps16* ^a^, *rps18*, *rps19*
RNA polymerase	*rpoA*, *rpoB*, *rpoC1* ^a^, *rpoC2*
Subunit of photosystem I	*psaA*, *psaB*, *psaC*, *psaI*, *psaJ*
Subunit of photosystem II	*psbA*, *psbB*, *psbC*, *psbD*, *psbE*, *psbF*, *psbH*, *psbI*, *psbJ*, *psbK*, *psbL*, *psbM*, *psbN*, *psbT*, *psbZ*
Subunit of cytochrome	*petA*, *petB* ^a^, *petD* ^a^, *petG*, *petL*, *petN*
Subunit of ATP synthases	*atpA*, *atpB*, *atpE*, *atpF* ^a^, *atpH*, *atpI*
Large unit of Rubisco	*rbcL*
Subunit of NADH dehydrogenase	*ndhA* ^a^, *ndhB* ^a^, *ndhC*, *ndhD*, *ndhE*, *ndhF*, *ndhG*, *ndhH*, *ndhI*, *ndhJ*, *ndhK*
Maturase	*matK*
Envelope membrane protein	*cemA*
Subunit of acetyl‐CoA	*accD*
C‐type cytochrome synthesis gene	*ccsA*
ATP‐dependent protease subunit P	*clpP* ^b^
Hypothetical proteins and conserved reading frames	*ycf1*, *ycf2*, *ycf3* ^b^, *ycf4*

^a^ Genes with single intron.

^b^ Genes with two introns.

### 3.2 Complete Chloroplast Genomes of *Gleditsia* Species

The cp genomes of the genus *Gleditsia* exhibited size variation. Specifically, *G. japonica* var. *delavayi* and *G. microphylla* both exceeded 170 kb, while the cp genomes of the other species were primarily in the range of 160–170 kb (Table 1). The genomes exhibited identical gene numbers and arrangements, suggesting that the cp genomes of this genus have preserved core functional stability throughout evolution. The genus *Gleditsia* showed a notable conservatism in GC content, ranging from 33.9% to 35.6%.

**FIGURE 2:**
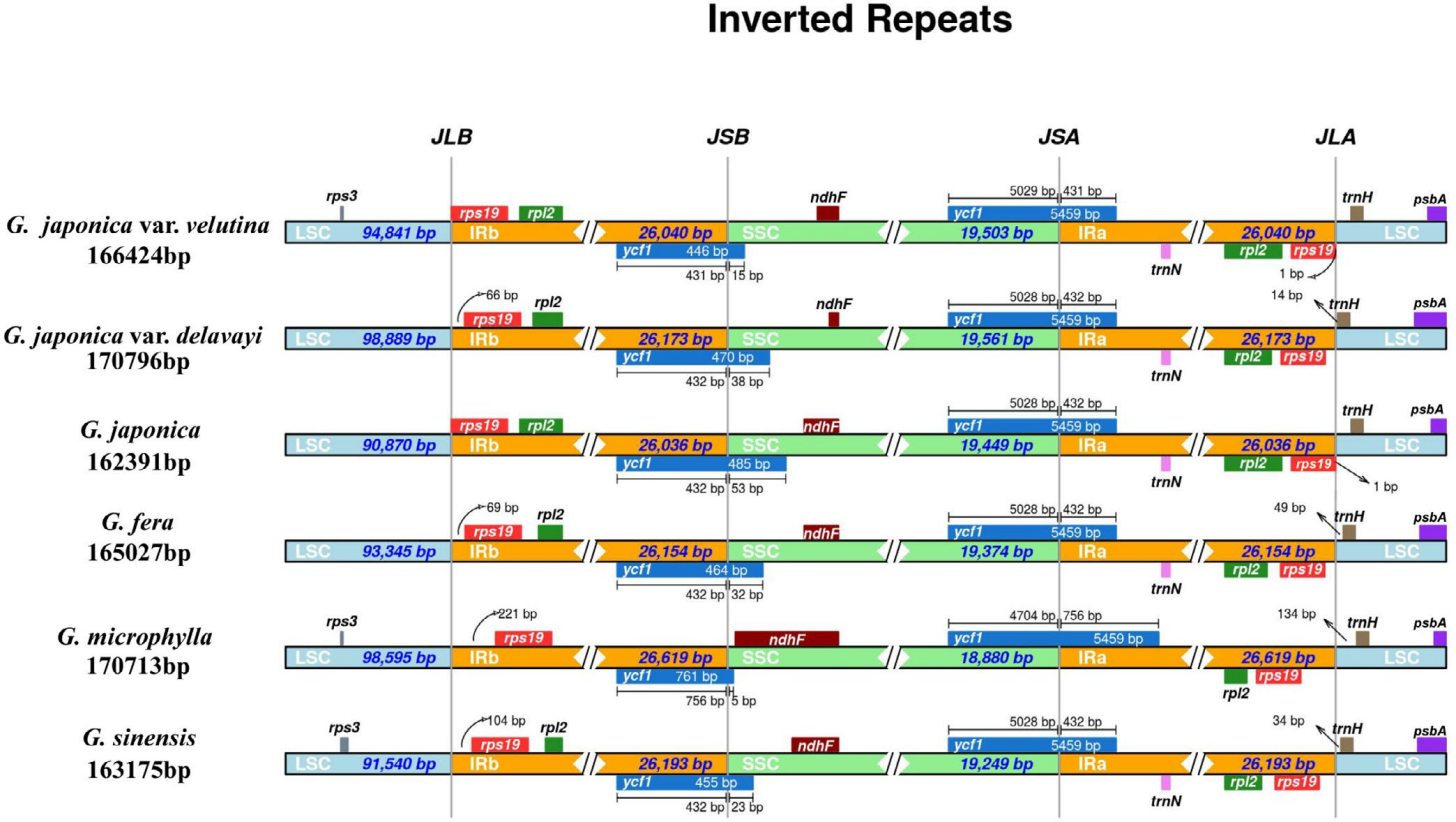
The comparison of the LSC, IR, and SSC border regions among six *Gleditsia* chloroplast genomes.

The expansion and contraction of the IR regions can affect chloroplast genome structure and may promote the evolution of gene duplication and functional divergence (Park et al. 2018; Wang et al. 2018), serving as a significant factor in chloroplast genome variation. The chloroplast genomes of *Gleditsia* species exhibited a conserved quadripartite structure, with relatively limited variation in IR length (Figure 2). Among the analyzed species, *G. microphylla* had the largest IR region, reaching 26,619 bp, whereas *G. japonica* had the smallest IR region at 26,036 bp. Similarly, *G. japonica* var. *velutina* and *G. japonica* var. *delavayi* also showed relatively small IR regions, with lengths of 26,040 and 26,173 bp, respectively. The comparison of IR/SC junctions revealed that IR boundary shifts resulted in the complete inclusion of *rps19* within the IR region in some taxa, whereas *rps19* was located at the IR/LSC boundary in *G. japonica* and *G. japonica* var. *velutina*. Overall, the IR regions were highly conserved within *Gleditsia*, suggesting that IR expansion and contraction contributed only slightly to chloroplast genome size variation. Instead, variation in the LSC region appeared to be the major factor underlying differences in chloroplast genome size among these species (Figure S1).

### 3.3 Analysis of Codon Preference

**FIGURE 3:**
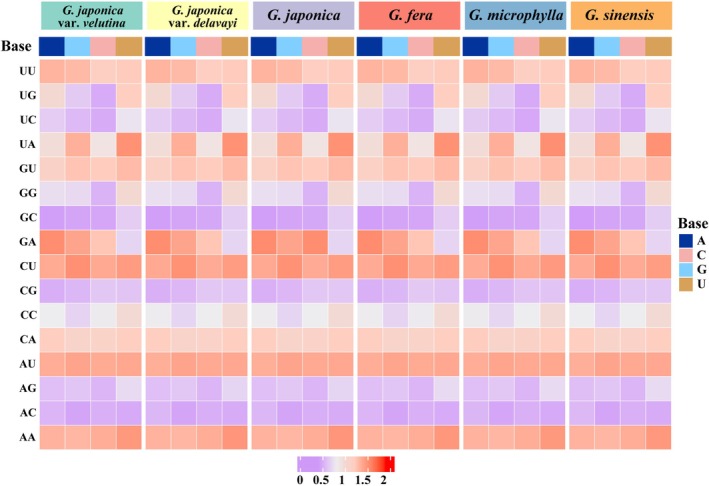
Codon usage bias analysis of chloroplast genome coding regions in six *Gleditsia* species. The labels display the first base of the species‐specific codons, while the left ordinate represents the second and third bases of the codons.

The synonymous codon usage patterns of six *Gleditsia* chloroplast genomes were analyzed based on RSCU values. The six species exhibited remarkably similar codon usage patterns for most amino acids (Figure 3 and Table S1). The preferred codons, defined as codons with RSCU values exceeding 1, were largely consistent among the six species. In addition, 30 optimal codons were consistently identified across the six *Gleditsia* species according to the criteria of RSCU > 1 and ΔRSCU > 0.08 (Table S2).

The SCUO analysis showed that the codon preference bias among the six *Gleditsia* species was notably similar, with all values falling below the median of 0.5, reflecting a consistently weak bias. The MILC value remained at 0.55, suggesting that the majority of genes in the chloroplast genome exhibited moderate expression strength. SCUO and MILC exhibited a weak positive correlation across all six species (*r* = 0.22–0.25, *p* < 0.05), indicating a restricted regulatory influence of codon usage preference on gene expression efficiency (Table 3). The *psa* (photosystem I) and *psb* (photosystem II) gene families exhibited high SCUO values (> 0.40) along with high MILC values (> 0.50), indicating that the bias was substantial in genes encoding core photosynthetic subunits (Table S3).

**TABLE 3:** SCUO, MILC values and correlation of six *Gleditsia* species.

Species	SCUO	MILC	*r*	*p*
*G. japonica* var. *velutina*	0.298	0.55	0.227	0.000
*G. japonica* var. *delavayi*	0.298	0.551	0.228	0.000
*G. japonica*	0.298	0.551	0.234	0.000
*G. fera*	0.298	0.551	0.227	0.000
*G. microphylla*	0.299	0.553	0.246	0.000
*G. sinensis*	0.299	0.552	0.210	0.000

Abbreviations: MILC, measures independent of length and composition; SCUO, synonymous codon usage order.

**FIGURE 4:**
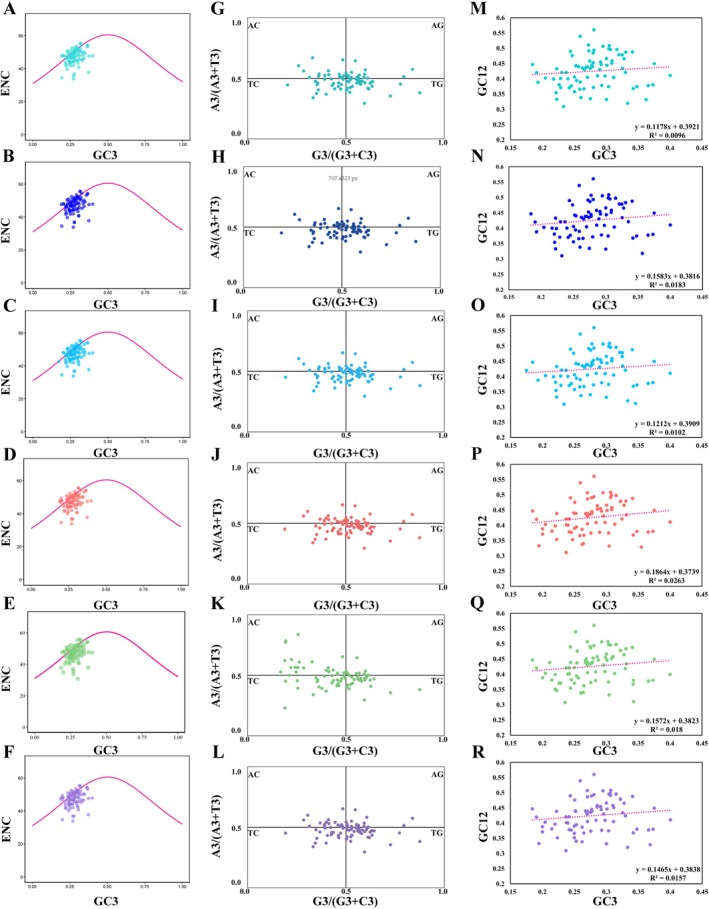
Codon usage bias analysis of chloroplast genome coding region in *Gleditsia*. From top to bottom is *G. japonica* var. *velutina*, *G. japonica* var. *delavayi*, *G. japonica* , *G. fera*, *G. microphylla* , *G. sinensis* . (A–F) The ENC‐plots of *Gleditsia*. (G–L) The PR2‐plots of *Gleditsia*. (M–R) The neutral plots of *Gleditsia*.

The ENC‐plot showed that most genes were below the standard curve (actual ENC value<anticipated value), with only a small percentage above the curve (Figure 4A–F). In the PR2 plot, most points deviated slightly from the central position, indicating an imbalance in A/T and G/C usage at the third codon position. This deviation suggests that synonymous codon usage in *Gleditsia* chloroplast genomes may be influenced by both mutational pressure and natural selection (Figure 4G–L). Based on the regression slopes of the neutrality plots, natural selection was estimated to contribute 81.36%–88.22% to codon usage bias, whereas mutational pressure contributed 11.78%–18.64% (Figure 4M–R).

### 3.4 Repeat Analysis

**FIGURE 5:**
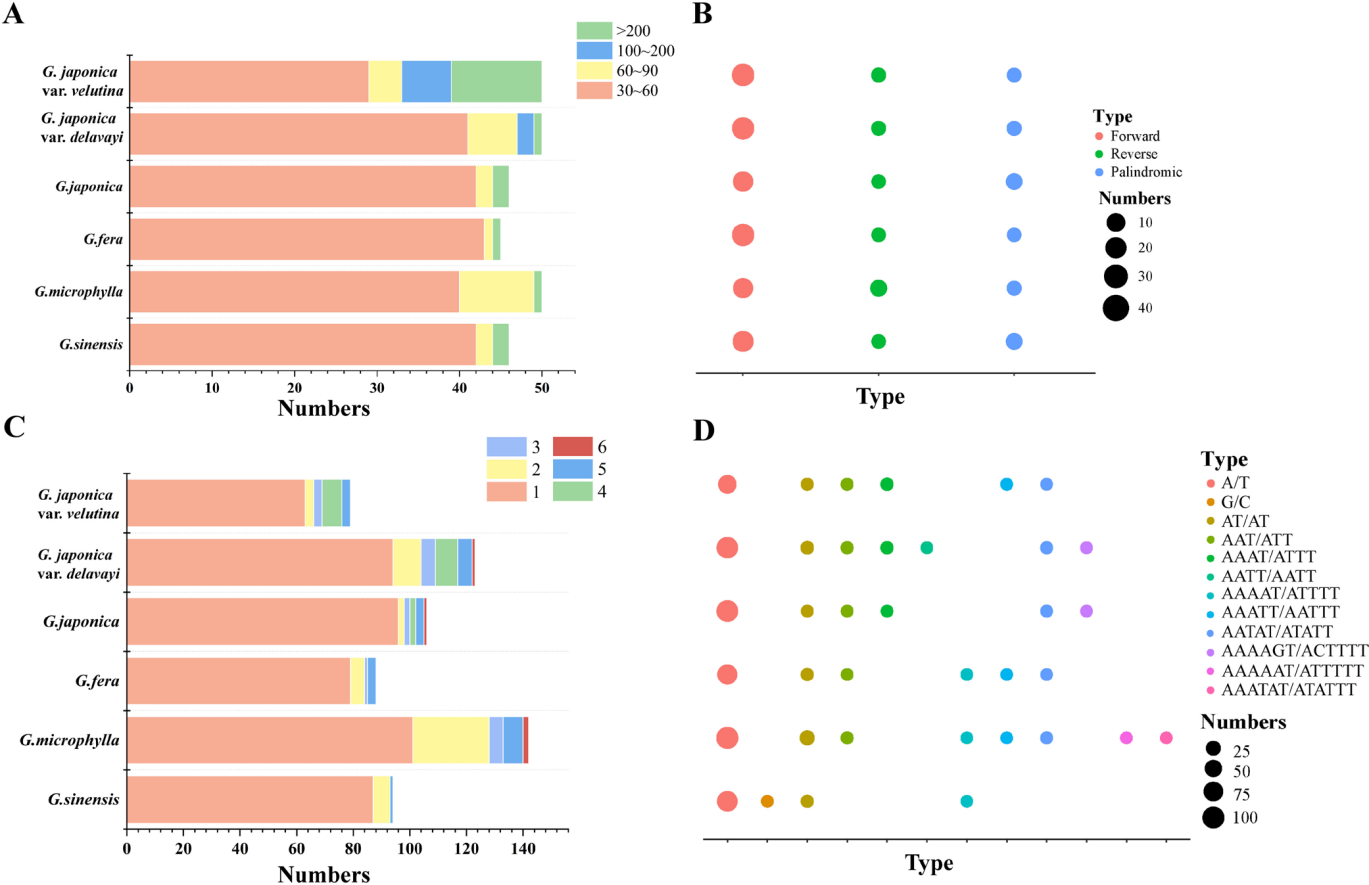
Analysis of dispersed repeats and simple sequence repeats (SSRs) in the chloroplast genomes of six *Gleditsia* species. (A, C) Number of repeats of different lengths. (B, D) Number of repeats by types.

Variation in repeat sequences arises from natural selection and adaptation to diverse environmental pressures (Britten and Kohne 1968), serving as a valuable resource for investigating population genetic structures and biogeographic histories of related taxa (Yang et al. 2016). The chloroplast genomes of the six *Gleditsia* species contained three types of dispersed repeats, namely forward, reverse, and palindromic repeats, with most repeats ranging from 30 to 60 bp in length. The length distribution of dispersed repeats showed that *G. japonica* var. *velutina* contained the highest number of long dispersed repeats longer than 200 bp, whereas repeats of 100–200 bp were mainly detected in *G. japonica* var. *velutina* and *G. japonica* var. *delavayi* (Figure 5A). Forward repeats were the predominant type in all species, whereas reverse and palindromic repeats showed interspecific variation. Among the six species, *G. microphylla* contained the highest number of reverse repeats (*n* = 15), while *G. japonica* and *G. sinensis* showed relatively high numbers of palindromic repeats (*n* = 13 each) (Figure 5B).

SSRs with mono‐ to hexanucleotide motifs were detected in the six *Gleditsia* chloroplast genomes. Mononucleotide SSRs were the most abundant type in all species, followed by dinucleotide and trinucleotide repeats, whereas tetra‐, penta‐, and hexanucleotide SSRs occurred at relatively low frequencies (Figure 5C). The motif composition analysis further showed that A/T mononucleotide repeats dominated the SSR profiles of all six species, indicating a strong A/T bias in chloroplast SSRs within *Gleditsia*. *G. microphylla* showed the highest abundance of A/T mononucleotide SSRs, whereas one C/G mononucleotide SSR was detected only in *G. sinensis* (Figure 5D).

All repeat sequences were identified as being enriched in the LSC and non‐coding regions (Table 4). A comparative examination of coding‐region SSRs showed significant conservation among four species ( *G. japonica* var. *delavayi*, *G. japonica* , *G. fera*, and *G. sinensis* ), with SSRs consistently positioned within eight core photosynthetic/metabolic genes: *atpB*, *ndhF*, *psbI*, *rpoB*, *rpoC2*, *rps18*, *ycf1*, and *ycf4* (Table S4). Both *ndhF* and *ycf1* exhibited SSRs across all six species analyzed. *G. japonica* var. *velutina* and *G. microphylla* shared similar SSR distributions, characterized by an equal total count of SSRs and congruent SSR motifs at 14 homologous loci. The expansion of the LSC region in *G. microphylla* led to the relocation of many SSRs to neighboring intergenic regions, without altering motif types or numbers in comparison to *G. japonica* var. *velutina*, indicating shared evolutionary constraints between these taxa.

**TABLE 4:** Tandem repeats and dispersed repeats in the chloroplast genomes of six *Gleditsia* species.

Type	Length	*G. japonica* var. *velutina*	*G. japonica* var. *delavayi*	*G. japonica*	*G. fera*	*G. microphylla*	*G. sinensis*
Tandem repeats sequence	< 100	76	72	76	71	68	81
	100–500	28	33	43	48	42	38
	500–1000	11	10	1	1	7	1
	> 1000	5	5	0	0	3	0
	LSC	120	120	120	120	120	120
Dispersed repeats sequence	30–60	29	41	42	43	40	42
	60–100	4	6	2	1	9	2
	100–200	6	2	0	0	0	0
	> 200	11	1	2	1	1	2
	LSC	43	42	36	43	44	43
	SSC	3	3	4	4	4	3
	IR	4	5	4	3	2	3
Simple repeats sequence	LSC	36	75	75	49	41	56
	SSC	21	19	19	21	6	19
	IR	7	4	4	4	17	5
	Coding Region	46	14	42	40	45	45
	IGS	18	84	56	34	19	35

In light of the strong association between LSC expansion and the increase of the total chloroplast genome size in the genus *Gleditsia* (Figure S1), we conducted a correlation analysis on three categories of repeat sequences prevalent in the LSC area (Figure S2). The findings indicate that the accumulation of tandem repeats in the LSC region directly facilitates its extension and is significantly associated with the expansion of the chloroplast genome.

### 3.5 Genome Sequence Divergence

**FIGURE 6:**
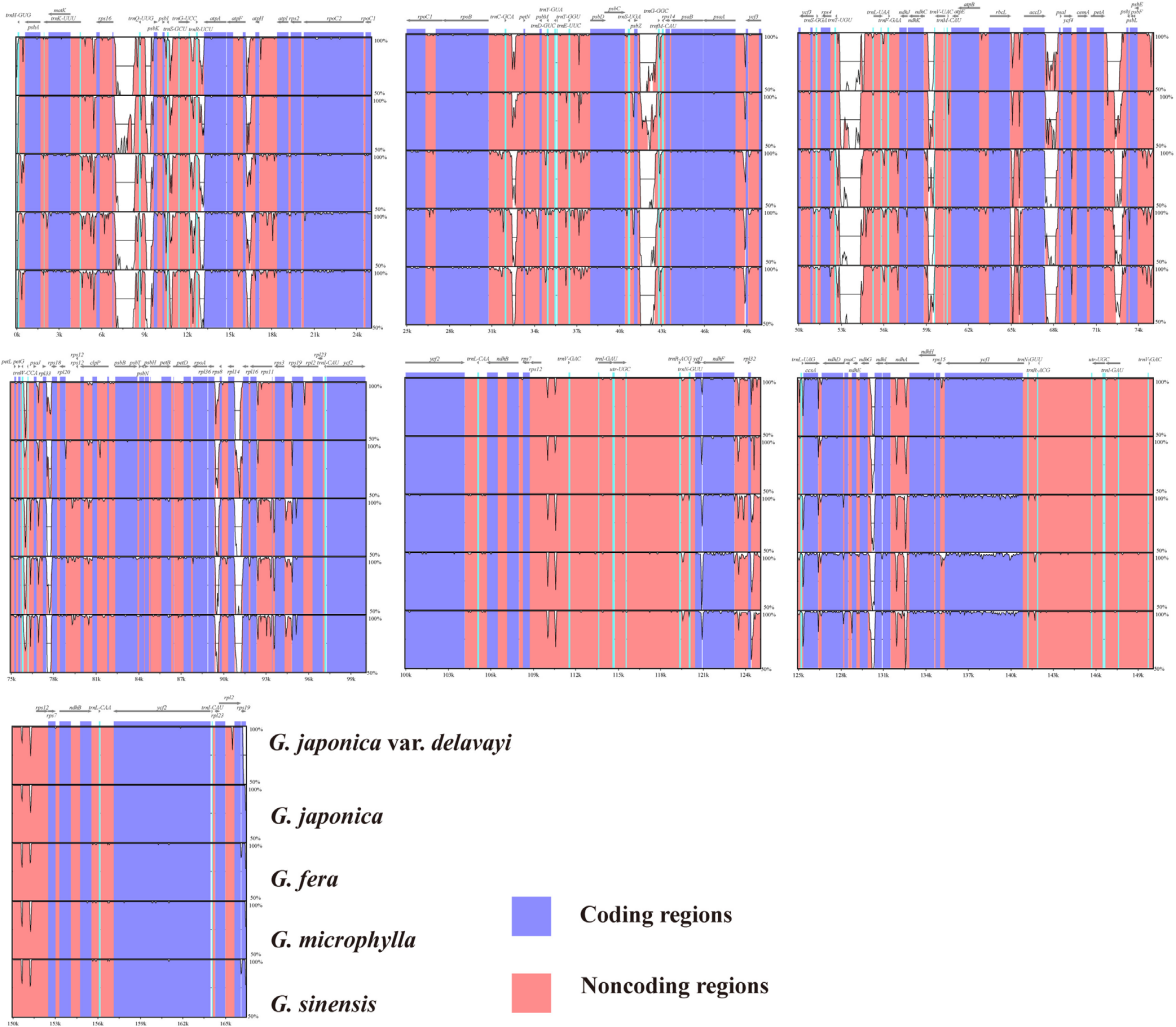
Visual comparison of chloroplast genomes of six *Gleditsia* species. The *y*‐axis represents the identity ranging from 50% to 100%, with coding regions marked in purple, non‐coding regions marked in pink, and untranslated regions (UTRs) in blue.

**FIGURE 7:**
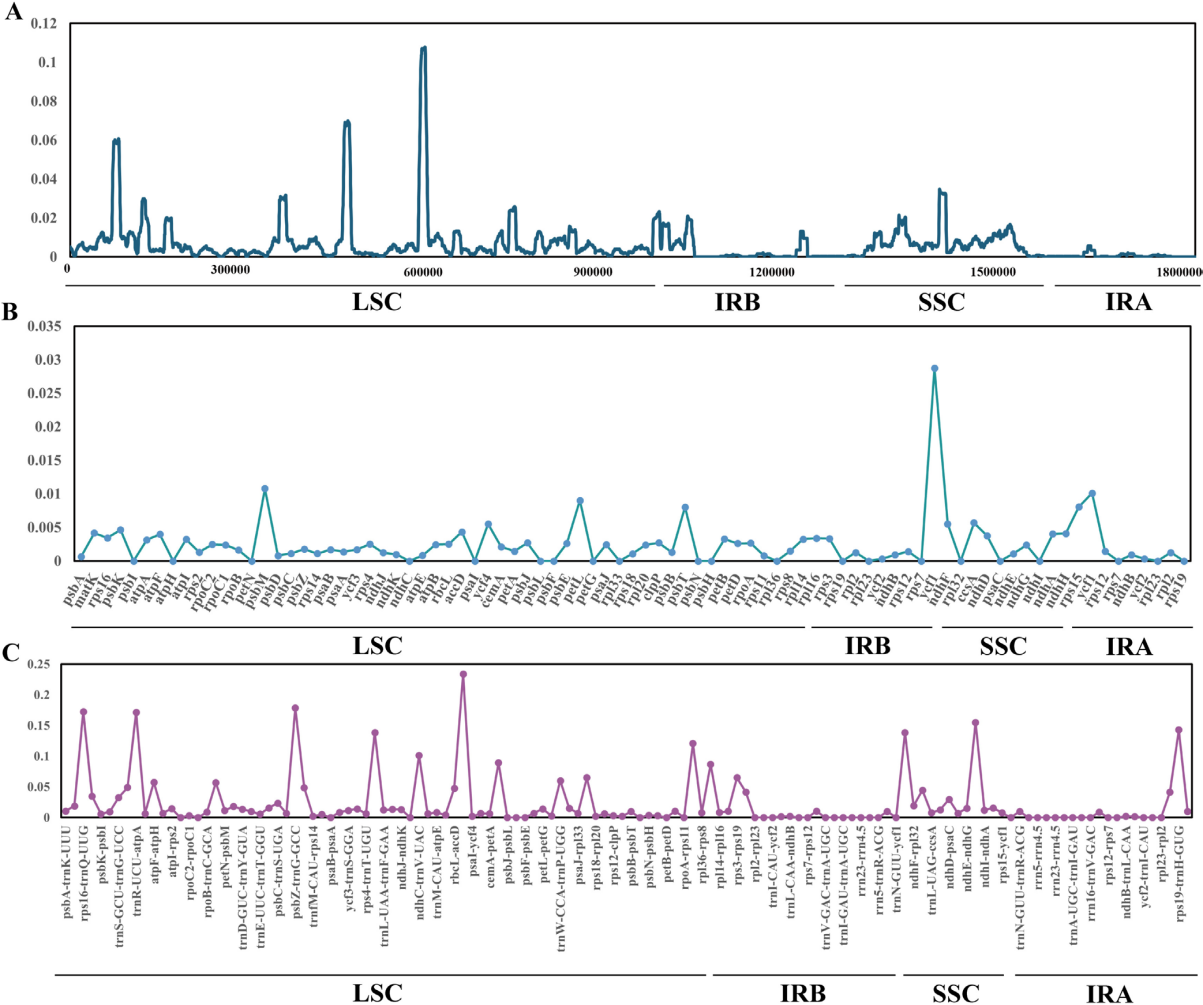
Nucleotide diversity (Pi) analysis of chloroplast genomes across six *Gleditsia* species. (A) Comparison of the Pi values of *Gleditsia* cp genome measured with a window of 2000 bp and a step size of 25 bp. (B) Comparison of Pi values in the genome coding region of *Gleditsia* cp. (C) Comparison of Pi values in the non‐coding region of *Gleditsia* cp genome.

Comparative analysis using mVISTA revealed that sequence variation among the six *Gleditsia* chloroplast genomes was mainly concentrated in the single‐copy regions, whereas the IR regions were relatively conserved (Figure 6). Nucleotide diversity (Pi) analysis further quantified sequence polymorphism across the chloroplast genomes. The IR region showed the lowest average Pi value (0.001), indicating a higher level of sequence conservation than the LSC and SSC regions, which had average Pi values of 0.008 and 0.011, respectively (Figure 7A). Overall, non‐coding regions exhibited substantially higher nucleotide diversity than coding regions, with mean Pi values of 0.025 and 0.002, respectively. In the coding regions, *ycf1* showed the highest nucleotide diversity (Pi = 0.029), possibly associated with variation near the IR/SC boundary, followed by *psbM* (Pi = 0.011) and *petL* (Pi = 0.010) (Figure 7B). In the non‐coding regions, the highly variable IGS regions identified by Pi analysis included *accD*–*psaI*, *rps16*–*trnQ‐UUG*, *psbZ*–*trnG‐GCC*, *trnR‐UCU*–*atpA*, and *trnT‐UGU*–*trnL‐UAA* (Figure 7C).

**FIGURE 8:**
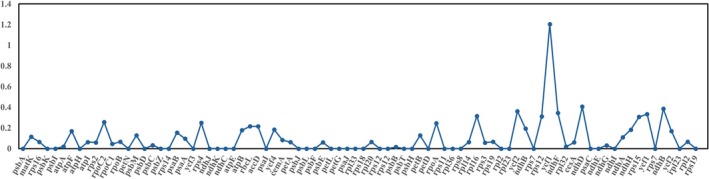
Selective pressure analysis (Ka/Ks) of chloroplast genomes among six *Gleditsia* species. *G. japonica* var. *velutina* was used as a reference for comprehensive comparison of Ka/Ks ratios with the other five species: *G. japonica* var. *delavayi*, *G. japonica* , *G. fera*, *G. microphylla* and *G. sinensis* .

Ka/Ks analysis by comparison of *G. japonica* var. *velutina* with the other five species showed that *ycf1* was the only gene that showed a positive selection signal (Ka/Ks > 1) in all five sets of comparisons, and the rest of the genes were subjected to purifying selection (Ka/Ks < 1) (Figure 8).

To further resolve the interspecific sequence variation patterns within the genus *Gleditsia*, single nucleotide polymorphism (SNP) analysis was carried out in genome‐wide comparisons using the *G. japonica* var. *velutina* cp genome as a reference. The results revealed a total of 389–1522 SNP sites, 293–482 insertion/deletion variants (indels), and 114–302 amino acid alterations across the five species, highlighting significant sequence diversity. Regional distribution analysis showed that most variants were concentrated in the LSC region, with fewer variants detected in the SSC and IR regions (Table 5).

**TABLE 5:** Comparative analysis of sequence variation among *Gleditsia* species, with *G. japonica* var. *velutina* as a reference.

SNP and indel	*G. japonica* var. *delavayi*	*G. japonica*	*G. fera*	*G. microphylla*	*G. sinensis*
Number					
SNP					
Transition	63	321	312	477	319
Transversion	589	68	831	1045	724
Total	652	389	1143	1522	1043
Insertion	114	131	261	188	281
Deletion	260	162	204	294	167
Substitution	169	114	204	302	184
Region					
SNP					
LSC	568	311	924	1149	823
SSC	56	53	182	200	166
IR	28	25	137	173	54
Insertion					
LSC	98	99	234	150	252
SSC	14	21	22	26	20
IR	2	11	5	12	9
Deletion					
LSC	212	137	166	254	133
SSC	21	17	23	23	14
IR	27	8	15	17	20
Substitution					
LSC	151	100	165	258	150
SSC	14	12	24	32	20
IR	4	2	15	12	14

### 3.6 Phylogenetic Analysis

**FIGURE 9:**
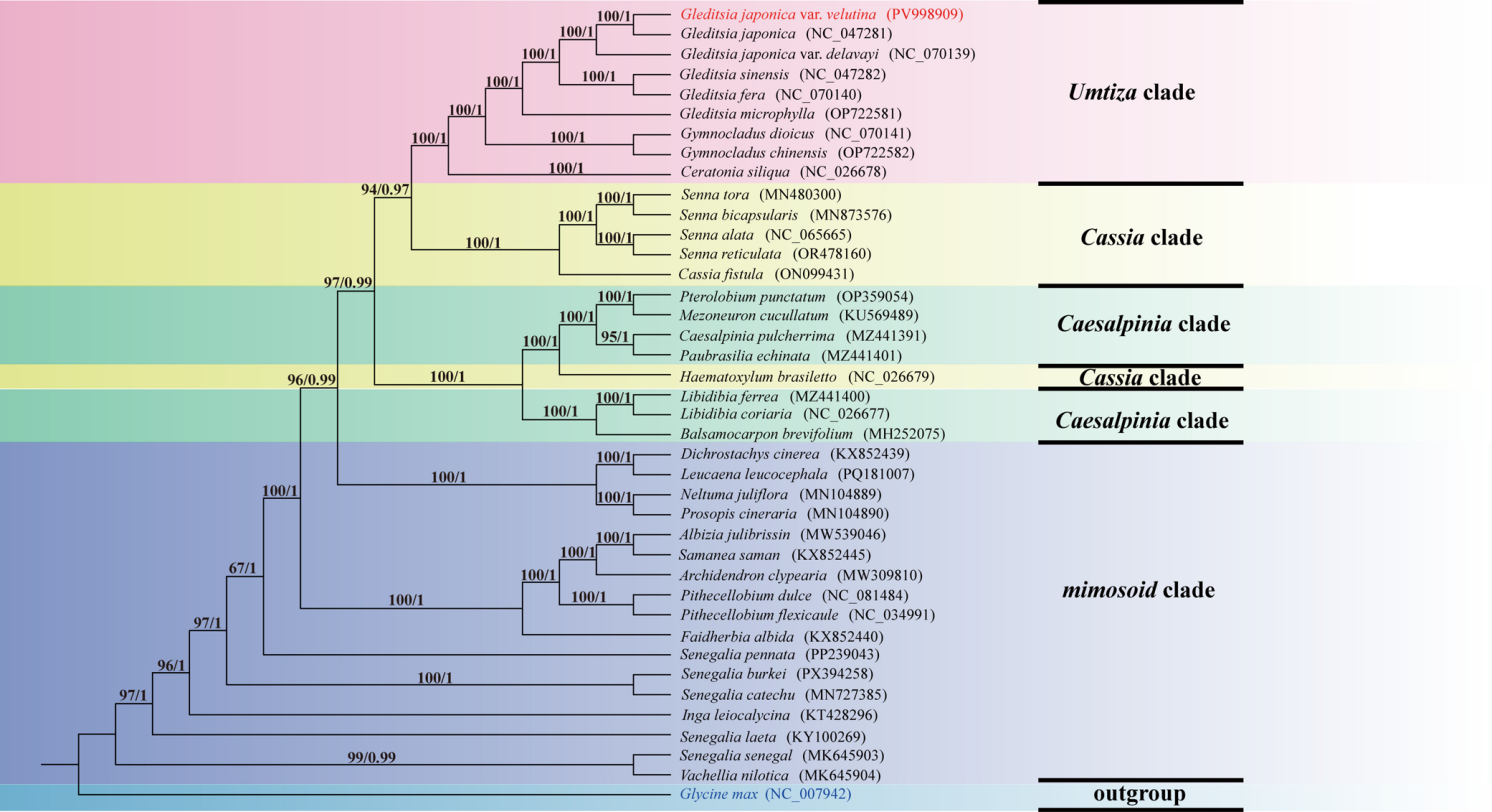
Phylogenetic tree of Caesalpinioideae based on the complete chloroplast genomes. The red indicates *Gleditsia japonica* var. *velutina*.

Phylogenetic analysis based on complete chloroplast genome sequences showed that the major clades within Caesalpinioideae were strongly supported (Figure 9). In the rooted topology, the *Umtiza* clade occupied an early‐branching position relative to the other sampled Caesalpinioideae clades. Within the *Umtiza* clade, *Ceratonia siliqua* was placed outside the strongly supported sister relationship between *Gymnocladus* and *Gleditsia*. Both *Gymnocladus* and *Gleditsia* were recovered as monophyletic genera, and these two genera were resolved as sister groups with high support values. Within *Gleditsia*, *G. japonica* , *G. japonica* var. *velutina*, and *G. japonica* var. *delavayi* clustered together, indicating their close relationship. *G. fera* and *G. sinensis* formed another clade, whereas *G. microphylla* was separated from the *G. fera*, *G. sinensis* clade.

## 4 Discussion

Although a reference chloroplast genome for *G. japonica* var. *velutina* has been published in the NCBI (OP764692), it remains incomplete due to challenges in resolving long repeat regions. Such complex structures often prevent short‐read sequencing data alone from assembling into a complete circular molecule. To address this limitation and obtain a complete chloroplast genome, we employed a hybrid sequencing strategy, integrating Oxford Nanopore long‐read sequencing, which can bridge repeat regions, with Illumina short‐read sequencing to ensure sequence accuracy. This approach aims to provide high‐quality genomic resources for accurate species identification, critical for guiding future conservation efforts.

### 4.1 Chloroplast Sequences Variation in *Gleditsia*

The cp genome of *G. japonica* var. *velutina* showed a highly conserved circular quadripartite structure corresponding with other species of *Gleditsia*, containing LSC, SSC, and IRs (Wu et al. 2024; Oyebanji et al. 2020; Zhang et al. 2023). The contents of A/T bases also exhibited a conserved pattern, aligning with that of other angiosperm chloroplast genomes (Zhang et al. 2023). However, the genome size exhibited some variation among the six species (162,391–170,796 bp; a difference of ~8 kb). Contrary to prevalent ideas attributing plant cp genome expansion to IR border changes (Li et al. 2020; Zheng et al. 2017), *Gleditsia* IR regions showed unique conservation in length and sequence across species, contributing minimally to size variance. Expansion was instead propelled by dynamic repeat accumulation in the LSC region resulting from the aggregation of repeat sequences. This repeat‐driven expansion mechanism has been observed in *Cypripedium* and *Campylotropis* (Feng et al. 2022; Bennetzen et al. 2005; Guo et al. 2021).

Repetitive sequences are a major driving force in genome evolution, and their accumulation is often associated with genomic rearrangements and sequence divergence (Britten and Kohne 1968; Britten and Davidson 1971). The six *Gleditsia* chloroplast genomes harbored numerous repetitive sequences, which were mainly distributed in the LSC and non‐coding regions, suggesting that these regions may contain informative polymorphisms suitable for future population‐level studies. Additionally, repeats in coding regions can drive the formation of new genes (Elder Jr. and Turner 1995). In *Gleditsia*, repeats located in coding areas predominantly comprise mononucleotide (A/T) simple sequence repeats (SSRs). These SSRs showed a notable level of conservation across the six species, occurring consistently within eight photosynthesis and metabolism‐related genes: *atpB*, *ndhF*, *psbI*, *rpoB*, *rpoC2*, *rps18*, *ycf1*, and *ycf4*. Furthermore, the abundant long dispersed repeats detected in *G. japonica* var. *velutina* may provide additional structural features for distinguishing this endangered taxon from closely related *Gleditsia* taxa.

Nucleotide substitutions and indels drive sequence divergence (Asano et al. 2004; Cavalier 2002), offering vital insights into molecular evolution (Muse and Gaut 1994; Britten et al. 2003). In *Gleditsia*, SNPs represented the commonest variation type, with a relatively high rate of transversion. Although DNA sequence evolution generally favors transitions, the A/T‐rich cp genome may exhibit elevated transversion frequencies (Yang and Yoder 1999; Morton and Clegg 1995; Morton et al. 1997). Notably, a transition preference was observed exclusively in *G. japonica* and its varieties ( *G. japonica* var. *delavayi*, *G. japonica* var. *velutina*). This conserved mutation bias, combined with minimal genetic divergence among the three taxa, supports their shared evolutionary trajectory (Fitch 1967; Li et al. 1985).

### 4.2 Codon Usage Bias and Taxonomic Implications

In addition to structural variations, patterns of sequence evolution can be examined through codon usage bias (CUB), which reflect selective pressures on gene function. Although CUB is a fundamental feature of genome evolution, previous *Gleditsia* cp genome studies treated it merely as supplementary evidence for divergence analysis (Xiao et al. 2024; Tan et al. 2020). In this study, we found highly conserved synonymous codon usage patterns across all six species. Our analyses further revealed a pronounced A/T bias driven predominantly by natural selection, consistent with the evolutionary features observed in Asteraceae (Kadam et al. 2024), *Hevea* (Li 1982), and *Pisum sativum* (Bhattacharyya et al. 2019). These patterns support the adaptive hypothesis of optimized A/T enrichment in higher plant chloroplast genomes (Sharp et al. 1988). However, CUB exerted only a weak regulatory influence on gene expression in *Gleditsia*, a phenomenon also seen in Theaceae (Wang et al. 2022), likely attributable to the high degree of evolutionary conservation of *Gleditsia*.

These evolutionary patterns provide new insights into long‐standing taxonomic controversies within *Gleditsia*. The classification of Asian *Gleditsia* taxonomy remains debated: Gordon classified *G. japonica* var. *delavayi* as a distinct species (*G. delavayi*), but Li reclassified it as a *G. japonica* variety and proposed a new variety *G. japonica* var. *velutina* (Li 1982; Gordon 1966), which is the currently prevalent taxonomic framework. The phylogenomic analysis of the Caesalpinioideae subfamily reveals that these three taxa form a monophyletic clade, clearly separated from other species in the *Umtiza* clade (Zhao et al. 2021). Unlike previous study by Xiao et al. (2024), our phylogenetic analysis supports the varietal relationship of *G. japonica* var. *delavayi* and *G. japonica* var. *velutina*. Furthermore, *G. fera* and *G. sinensis* consistently form a distinct sister clade, aligning with the intrageneric evolutionary model proposed by Tan et al. (2020). The high similarity in their patterns of genetic variation suggests a recent common ancestry with limited genomic differentiation.

Precise identification of closely related taxa is essential for effective conservation, and DNA barcoding offers a reliable tool for this purpose (Zhou et al. 2016; Li et al. 2015), particularly for the endangered *G. japonica* var. *velutina* (Lin et al. 2022). Several highly variable IGS regions emerge as promising candidate markers for distinguishing closely related *Gleditsia* taxa. Among coding regions, *ycf1* showed the highest nucleotide diversity and was the only gene showing a positive selection signal, further supporting its potential utility as a plastid barcode. Its effectiveness as a universal land plant barcode has been demonstrated for interspecific delineation in Orchidaceae and Pinaceae (Li et al. 2015; Gernandt et al. 2009; Dong et al. 2015). In addition, *ndhF* also showed relatively high sequence divergence and Ka/Ks values, and its association with the highly variable *ycf1*–*ndhF* region suggests it may serve as another informative marker for *Gleditsia* identification.

## 5 Conclusion

In this study, we integrated Nanopore long‐read and Illumina short‐read sequencing to successfully assemble a high‐quality, complete chloroplast genome for *G. japonica* var. *velutina*. Comparative analyses with congeners revealed that the plastomes of *Gleditsia* exhibit a highly conserved quadripartite structure, with stable patterns of sequence variation and codon usage bias. Our results demonstrate that plastome expansion in *Gleditsia* is primarily driven by the accumulation of repetitive sequences in the LSC region, rather than IR boundary shifts. This provides new insights into the evolutionary mechanisms underlying chloroplast genome size variation. Codon usage pattern further exhibits a pronounced A/T bias shaped predominantly by natural selection, while exerting only a weak influence on gene expression. Phylogenomic analyses support the varietal status of *G. japonica* var. *delavayi* and *G. japonica* var. *velutina*, thereby effectively resolving long‐standing taxonomic controversies within Asian *Gleditsia*. Furthermore, we identified hypervariable intergenic regions and polymorphic genes as promising molecular markers for species identification and phylogenetic studies. These resources are particularly valuable for the conservation of the endangered *G. japonica* var. *velutina*. Overall, this study deepens our understanding of plastome evolution in *Gleditsia* and provides valuable genomic resources for future taxonomic, population genetic, and conservation studies.

## Author Contributions

**Shiyu Hu:** data curation (equal), methodology (equal), software (equal), visualization (equal), writing – original draft (equal). **Naiwei Li:** data curation (equal), investigation (equal), resources (equal), writing – review and editing (equal). **Xingjian Liu:** resources (equal). **Yueqi Sun:** resources (equal). **Baocheng Wu:** resources (equal). **Mimi Li:** conceptualization (equal), funding acquisition (equal), methodology (equal), resources (equal), supervision (equal), validation (equal), writing – review and editing (equal).

## Funding

This research was supported by the NBG Fund for Connotation Construction (NBGF202304) and the Scientific Fund of Nanjing Botanical Garden Mem. Sun Yat‐Sen (JSPKLB202505).

## Conflicts of Interest

The authors declare no conflicts of interest.

## Supporting information


**Figure S1:** Correlation between the dimensions of the chloroplast genome. (A) LSC, (B) SSC, (C) IRs.
**Figure S2:** Correlation between the dimensions of the chloroplast genome and (A) tandem repeat sequences, (B) dispersed repeat sequences, (C) SSRs. Only repeat sequences in the LSC were calculated.

**Table S1:** Relative synonymous codon usage (RSCU) values in *Gleditsia*.
**Table S2:** Optimal codons in *Gleditsia*.
**Table S3:** Codon preferences and gene expression levels in the *psb* gene of *Gleditsia*.
**Table S4:** Simple sequence repeats (SSRs) in coding regions of *Gleditsia*.

## Data Availability

The plastid genome has been deposited in GenBank (https://www.ncbi.nlm.nih.gov/) under the accession numbers: PV998909.
