# Supplementary material for: Chloroplast Genome Insights of Gleditsia japonica var. velutina and Evolutionary Implications for Variation and Phylogeny in Gleditsia
Source: Ecol Evol. 2026 Jun 11;16(6):e73826. doi: 10.1002/ece3.73826 (PMC13255012; doi:10.1002/ece3.73826)
Supplement: Supplementary file 1 — Figure S1: Correlation between the dimensions of the chloroplast genome. (A) LSC, (B) SSC, (C) IRs. Figure S2: Correlation between the dimensions of the chloroplast genome and (A) tandem repeat sequences, (B) dispersed repeat sequences, (C) SSRs. Only repeat sequences in the LSC were calculated. [file ECE3-16-e73826-s001.docx]

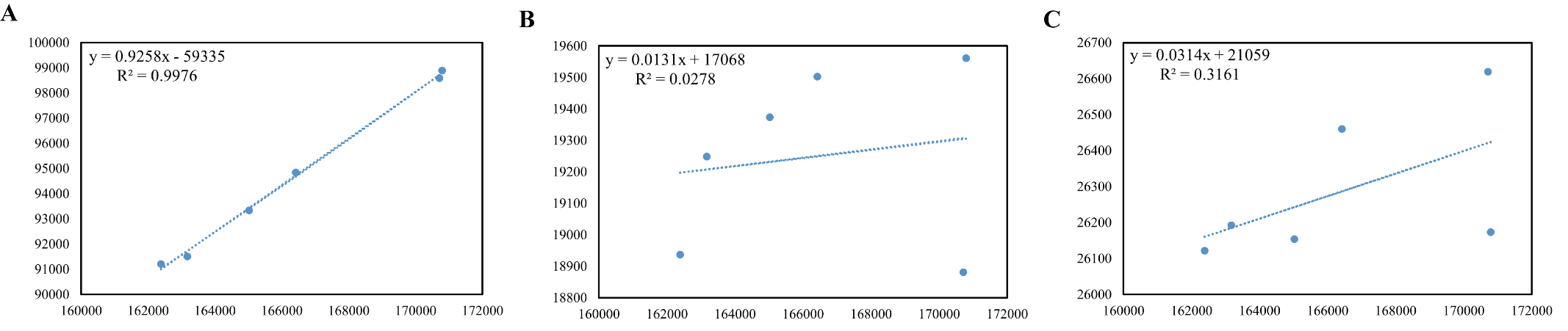


FIGURE S1. Correlation between the dimensions of the chloroplast genome. (A) LSC, (B) SSC, (C) IRS.


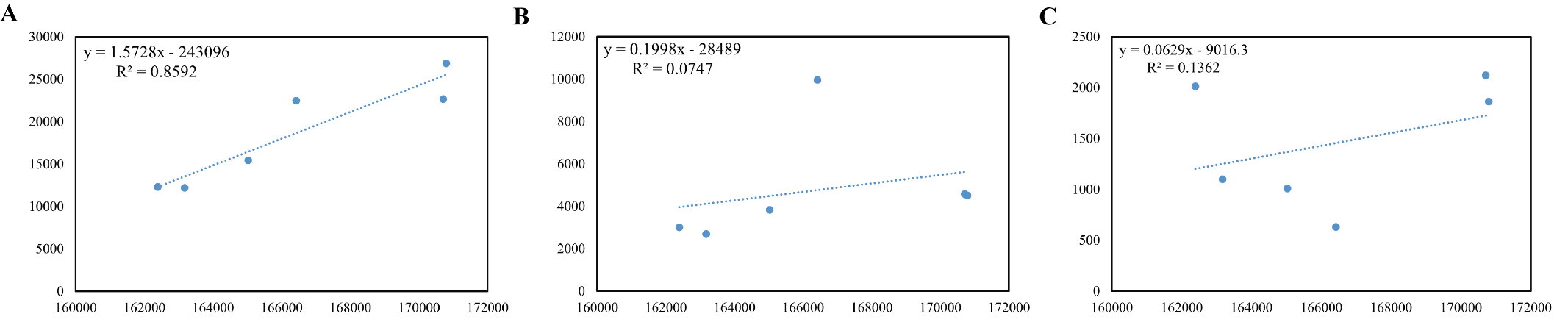


FIGURE S2. Correlation between the dimensions of the chloroplast genome. (A) tandem repeat sequences, (B) dispersed repeat sequences, (C) SSRs. Only repeat sequences in the LSC were calculated.
