# Supplementary material for: Chloroplast Genome Insights of Gleditsia japonica var. velutina and Evolutionary Implications for Variation and Phylogeny in Gleditsia
Source: Ecol Evol. 2026 Jun 11;16(6):e73826. doi: 10.1002/ece3.73826 (PMC13255012; doi:10.1002/ece3.73826)
Supplement: Supplementary file 2 — Table S1: Relative synonymous codon usage (RSCU) values in Gleditsia. Table S2: Optimal codons in Gleditsia. Table S3: Codon preferences and gene expression levels in the psb gene of Gleditsia. Table S4: Simple sequence repeats (SSRs) in coding regions of Gleditsia. [file ECE3-16-e73826-s002.docx]

TABLE S1. Relative synonymous codon usage (RSCU) values in *Gleditsia*.

| **Amino acid** | **Codon** | ***G. japonica* var. *velutina*** | ***G. japonica* var. *delavayi*** | ***G. japonica*** | ***G. fera*** | ***G. microphylla*** | ***G. sinensis*** |
| --- | --- | --- | --- | --- | --- | --- | --- |
| Phe | UUU | 1.312 | 1.312 | 1.314 | 1.315 | 1.303 | 1.313 |
|  | UUC | 0.688 | 0.688 | 0.686 | 0.685 | 0.697 | 0.687 |
| Ser | UCU | 1.717 | 1.719 | 1.714 | 1.712 | 1.732 | 1.706 |
|  | UCC | 0.977 | 0.973 | 0.976 | 0.971 | 0.968 | 0.976 |
|  | UCA | 1.271 | 1.273 | 1,278 | 1.27 | 1.261 | 1.269 |
|  | UCG | 0.517 | 0.518 | 0.513 | 0.524 | 0.512 | 0.529 |
|  | AGU | 1.179 | 1.172 | 1.174 | 1.182 | 1.184 | 1.183 |
|  | AGC | 0.339 | 0.345 | 0.344 | 0.341 | 0.343 | 0.337 |
| Tyr | UAU | 1.604 | 1.607 | 1.606 | 1.601 | 1.608 | 1.601 |
|  | UAC | 0.396 | 0.393 | 0.394 | 0.399 | 0.392 | 0.399 |
| Cys | UGU | 1.444 | 1.444 | 1.444 | 1.438 | 1.433 | 1.438 |
|  | UGC | 0.556 | 0.556 | 0.556 | 0.562 | 0.567 | 0.562 |
| Leu | UUA | 1.837 | 1.836 | 1.842 | 1.829 | 1.852 | 1.833 |
|  | UUG | 1.271 | 1.273 | 1.273 | 1.277 | 1.271 | 1.276 |
|  | CUU | 1.247 | 1.247 | 1.245 | 1.255 | 1.252 | 1.261 |
|  | CUC | 0.401 | 0.398 | 0.403 | 0.398 | 0.386 | 0.399 |
|  | CUA | 0.848 | 0.85 | 0.842 | 0.842 | 0.847 | 0.836 |
|  | CUG | 0.397 | 0.396 | 0.396 | 0.4 | 0.392 | 0.394 |
| Ter | UAA | 1.765 | 1.75 | 1.8 | 1.786 | 1.75 | 1.756 |
|  | UGA | 0.6 | 0.607 | 0.6 | 0.607 | 0.607 | 0.6 |
|  | UAG | 0.635 | 0.643 | 0.6 | 0.607 | 0.643 | 0.635 |
| Trp | UGG | 1 | 1 | 1 | 1 | 1 | 1 |
| Pro | CCU | 1.585 | 1.588 | 1.583 | 1.578 | 1.586 | 1.578 |
|  | CCC | 0.729 | 0.73 | 0.735 | 0.742 | 0.739 | 0.742 |
|  | CCA | 1.149 | 1.148 | 1.142 | 1.153 | 1.166 | 1.156 |
|  | CCG | 0.537 | 0.534 | 0.539 | 0.527 | 0.51 | 0.524 |
| His | CAU | 1.583 | 1.583 | 1.582 | 1.587 | 1.585 | 1.584 |
|  | CAC | 0.417 | 0.417 | 0.418 | 0.413 | 0.415 | 0.416 |
| Arg | CGU | 1.31 | 1.306 | 1.308 | 1.306 | 1.299 | 1.31 |
|  | CGC | 0.374 | 0.374 | 0.374 | 0.379 | 0.377 | 0.379 |
|  | CGA | 1.352 | 1.352 | 1.354 | 1.352 | 1.356 | 1.352 |
|  | CGG | 0.428 | 0.432 | 0.427 | 0.429 | 0.423 | 0.429 |
|  | AGA | 1.883 | 1.879 | 1.88 | 1.872 | 1.878 | 1.861 |
|  | AGG | 0.653 | 0.657 | 0.656 | 0.662 | 0.667 | 0.67 |
| Gln | CAA | 1.558 | 1.556 | 1.559 | 1.558 | 1.556 | 1.558 |
|  | CAG | 0.442 | 0.444 | 0.441 | 0.442 | 0.444 | 0.442 |
| Ile | AUU | 1.5 | 1.5 | 1.499 | 1.5 | 1.505 | 1.497 |
|  | AUC | 0.56 | 0.56 | 0.558 | 0.56 | 0.55 | 0.559 |
|  | AUA | 0.94 | 0.94 | 0.942 | 0.94 | 0.944 | 0.944 |
| Thr | ACU | 1.578 | 1.582 | 1.574 | 1.585 | 1.571 | 1.578 |
|  | ACC | 0.737 | 0.739 | 0.745 | 0.738 | 0.73 | 0.743 |
|  | ACA | 1.267 | 1.267 | 1.264 | 1.262 | 1.286 | 1.264 |
|  | ACG | 0.417 | 0.412 | 0.416 | 0.415 | 0.413 | 0.414 |
| Asn | AAU | 1.554 | 1.556 | 1.558 | 1.545 | 1.553 | 1.54 |
|  | AAC | 0.446 | 0.444 | 0.442 | 0.455 | 0.447 | 0.46 |
| Lys | AAA | 1.51 | 1.51 | 1.511 | 1.51 | 1.509 | 1.512 |
|  | AAG | 0.49 | 0.49 | 0.489 | 0.49 | 0.491 | 0.488 |
| Met | AUG | 1 | 1 | 1 | 1 | 1 | 1 |
| Val | GUU | 1.458 | 1.456 | 1.457 | 1.447 | 1.447 | 1.447 |
|  | GUC | 0.465 | 0.466 | 0.462 | 0.459 | 0.467 | 0.459 |
|  | GUA | 1.532 | 1.533 | 1.536 | 1.546 | 1.54 | 1.548 |
|  | GUG | 0.545 | 0.545 | 0.544 | 0.549 | 0.546 | 0.546 |
| Ala | GCU | 1.845 | 1.843 | 1.846 | 1.835 | 1.831 | 1.84 |
|  | GCC | 0.598 | 0.595 | 0.598 | 0.606 | 0.614 | 0.608 |
|  | GCA | 1.105 | 1.109 | 1.109 | 1.11 | 1.103 | 1.109 |
|  | GCG | 0.453 | 0.453 | 0.447 | 0.449 | 0.451 | 0.443 |
| Asp | GAU | 1.628 | 1.629 | 1.631 | 1.631 | 1.623 | 1.634 |
|  | GAC | 0.372 | 0.371 | 0.369 | 0.369 | 0.377 | 0.366 |
| Gly | GGA | 1.631 | 1.632 | 1.367 | 1.633 | 1.623 | 1.629 |
|  | GGU | 1.369 | 1.367 | 0.368 | 1.367 | 1.378 | 1.366 |
|  | GGG | 0.633 | 0.632 | 1,634 | 0.635 | 0.635 | 0.636 |
|  | GGC | 0.367 | 0.369 | 0.631 | 0.365 | 0.365 | 0.369 |
| Glu | GAA | 1.477 | 1.477 | 1.476 | 1.474 | 1.471 | 1.478 |
|  | GAG | 0.523 | 0.523 | 0.524 | 0.526 | 0.529 | 0.522 |

TABLE S2. Optimal codons in *Gleditsia*.

| **Amino acid** | ***G. japonica* var. *velutina*** | ***G. japonica* var. *delavayi*** | ***G. japonica*** | ***G. fera*** | ***G. microphylla*** | ***G. sinensis*** |
| --- | --- | --- | --- | --- | --- | --- |
| Phe | UUU | UUU | UUU | UUU | UUU | UUU |
| Ser | UCU | UCU | UCU | UCU | UCU | UCU |
|  | UCA | UCA | UCA | UCA | UCA | UCA |
|  | AGU | AGU | AGU | AGU | AGU | AGU |
| Tyr | UAU | UAU | UAU | UAU | UAU | UAU |
| Cys | UGU | UGU | UGU | UGU | UGU | UGU |
| Leu | UUA | UUA | UUA | UUA | UUA | UUA |
|  | UUG | UUG | UUG | UUG | UUG | UUG |
|  | CUU | CUU | CUU | CUU | CUU | CUU |
| Ter | UAA | UAA | UAA | UAA | UAA | UAA |
| Pro | CCU | CCU | CCU | CCU | CCU | CCU |
|  | CCA | CCA | CCA | CCA | CCA | CCA |
| His | CAU | CAU | CAU | CAU | CAU | CAU |
| Arg | CGU | CGU | CGU | CGU | CGU | CGU |
|  | CGA | CGA | CGA | CGA | CGA | CGA |
|  | AGA | AGA | AGA | AGA | AGA | AGA |
| Gln | CAA | CAA | CAA | CAA | CAA | CAA |
| Ile | AUU | AUU | AUU | AUU | AUU | AUU |
| Thr | ACU | ACU | ACU | ACU | ACU | ACU |
|  | ACA | ACA | ACA | ACA | ACA | ACA |
| Asn | AAU | AAU | AAU | AAU | AAU | AAU |
| Lys | AAA | AAA | AAA | AAA | AAA | AAA |
| Val | GUU | GUU | GUU | GUU | GUU | GUU |
|  | GUA | GUA | GUA | GUA | GUA | GUA |
| Ala | GCU | GCU | GCU | GCU | GCU | GCU |
|  | GCA | GCA | GCA | GCA | GCA | GCA |
| Asp | GAU | GAU | GAU | GAU | GAU | GAU |
| Gly | GGA | GGA | GGA | GGA | GGA | GGA |
|  | GGU | GGU | GGU | GGU | GGU | GGU |
| Glu | GAA | GAA | GAA | GAA | GAA | GAA |

TABLE S3. Codon preferences and gene expression levels in the *psb* genes of *Gleditsia*.

| **Gene** | ***G. japonica* var. *velutina*** | | ***G. japonica* var. *delavayi*** | | ***G. japonica*** | | ***G. fera*** | | ***G. microphylla*** | | ***G. sinensis*** | |
| --- | --- | --- | --- | --- | --- | --- | --- | --- | --- | --- | --- | --- |
|  | SCUO | MILC | SCUO | MILC | SCUO | MILC | SCUO | MILC | SCUO | MILC | SCUO | MILC |
| *PsaI* | 0.564 | 0.751 | 0.564 | 0.751 | 0.564 | 0.751 | 0.564 | 0.751 | 0.564 | 0.751 | 0.564 | 0.751 |
| *psaJ* | 0.449 | 0.650 | 0.449 | 0.650 | 0.449 | 0.650 | 0.449 | 0.650 | 0.439 | 0.636 | 0.449 | 0.650 |
| *psbE* | 0.389 | 0.666 | 0.393 | 0.710 | 0.393 | 0.710 | 0.389 | 0.703 | 0.393 | 0.710 | 0.393 | 0.710 |
| *psbF* | 0.444 | 0.514 | 0.444 | 0.514 | 0.444 | 0.514 | 0.444 | 0.514 | 0.444 | 0.514 | 0.444 | 0.514 |
| *psbH* | 0.49 | 0.586 | 0.490 | 0.586 | 0.490 | 0.586 | 0.490 | 0.586 | 0.490 | 0.586 | 0.490 | 0.586 |
| *psbI* | 0.581 | 0.847 | 0.581 | 0.847 | 0.581 | 0.847 | 0.581 | 0.847 | 0.581 | 0.847 | 0.581 | 0.847 |
| *psbJ* | 0.553 | 0.613 | 0.553 | 0.613 | 0.553 | 0.613 | 0.553 | 0.613 | 0.553 | 0.613 | 0.553 | 0.613 |
| *psbM* | 0.517 | 0.508 | 0.517 | 0.508 | 0.517 | 0.508 | 0.530 | 0.499 | 0.530 | 0.537 | 0.530 | 0.499 |
| *psbN* | 0.448 | 0.506 | 0.448 | 0.506 | 0.448 | 0.506 | 0.448 | 0.506 | 0.448 | 0.506 | 0.448 | 0.506 |

TABLE S4. Simple sequence repeats (SSRs) in coding regions of *Gleditsia*.

| **Species** | **Type** | **Star** | **End** | **Gene name** | **Species** | **Type** | **Star** | **End** | **Gene name** |
| --- | --- | --- | --- | --- | --- | --- | --- | --- | --- |
| ***G. japonica* var. *velutina*** | (AT)9 | 121916 | 121933 | *ndhF* | ***G. fera*** | (A)12 | 9796 | 9807 | *psbI* |
|  | (AATAT)3 | 135462 | 135476 | *ycf1* |  | (T)10 | 29656 | 29665 | *rpoB* |
|  | (T)12 | 62363 | 62374 | *atpB* |  | (T)10 | 61833 | 61842 | *atpB* |
|  | (T)12 | 66021 | 66032 | *accD* |  | (A)11 | 68264 | 68274 | *ycf4* |
|  | (A)12 | 87961 | 87972 | *rpoA* |  | (A)10 | 76939 | 76948 | *rps18* |
|  | (A)11 | 49936 | 49946 | *ycf3* |  | (T)14 | 120343 | 120356 | *ndhF* |
|  | (A)11 | 57851 | 57861 | *ndhK* |  | (T)12 | 134779 | 134790 | *ycf1* |
|  | (A)11 | 62573 | 62583 | *atpB* |  | (T)14 | 135015 | 135028 | *ycf1* |
|  | (A)11 | 78672 | 78682 | *rpl20* |  | (A)10 | 136027 | 136036 | *ycf1* |
|  | (T)11 | 100407 | 100417 | *ycf2* |  | (A)10 | 137155 | 137164 | *ycf1* |
|  | (A)11 | 120554 | 120564 | *ycf1* |  | (A)10 | 137735 | 137744 | *ycf1* |
|  | (A)11 | 120752 | 120762 | *ycf1* |  | (T)15 | 138504 | 138518 | *ycf1* |
|  | (T)11 | 122763 | 122773 | *ndhF* | ***G. microphylla*** | (A)10 | 51969 | 51978 | *rps4* |
|  | (A)11 | 133746 | 133756 | *ndhH* |  | (A)11 | 58864 | 58874 | *ndhK* |
|  | (A)10 | 51969 | 51978 | *rps4* |  | (A)10 | 61868 | 61877 | *atpE* |
|  | (A)10 | 61868 | 61877 | *atpB* |  | (T)12 | 62363 | 62374 | *atpB* |
|  | (A)10 | 64296 | 64305 | *rbcL* |  | (A)11 | 62573 | 62583 | *atpB* |
|  | (A)10 | 77367 | 77376 | *rpl33* |  | (A)11 | 62757 | 62767 | *atpB* |
|  | (T)10 | 83013 | 83022 | *psbB* |  | (A)10 | 64296 | 64305 | *rbcL* |
|  | (A)10 | 96728 | 96737 | *rpl23* |  | (T)10 | 76017 | 76026 | *psbE* |
|  | (A)10 | 98261 | 98270 | *ycf2* |  | (T)10 | 80163 | 80172 | *rps18* |
|  | (T)10 | 101810 | 101819 | *ycf2* |  | (A)11 | 80732 | 80742 | *rpl20* |
|  | (A)10 | 131265 | 131274 | *ndhA* |  | (T)10 | 83013 | 83022 | *clpP* |
|  | (T)10 | 135640 | 135649 | *ycf1* |  | (T)11 | 86442 | 86452 | *psbN* |
| ***G. japonica* var. *delavayi*** | (A)13 | 12680 | 12692 | *psbI* |  | (A)10 | 86807 | 86816 | *psbH* |
|  | (T)10 | 32936 | 32945 | *rpoB* |  | (A)12 | 87961 | 87972 | *petB* |
|  | (A)10 | 82004 | 82013 | *rps18* |  | (T)11 | 100407 | 100417 | *rpl2* |
|  | (T)14 | 125929 | 125942 | *ndhF* |  | (T)10 | 101810 | 101819 | *ycf2* |
|  | (T)12 | 140529 | 140540 | *ycf1* |  | (T)10 | 104345 | 104354 | *ycf2* |
|  | (T)14 | 140765 | 140778 | *ycf1* |  | (T)10 | 124905 | 124914 | *ycf1* |
|  | (A)10 | 141777 | 141786 | *ycf1* |  | (A)10 | 126557 | 126566 | *ndhF* |
|  | (T)10 | 142301 | 142310 | *ycf1* |  | (A)10 | 131265 | 131274 | *ndhD* |
|  | (A)10 | 142905 | 142914 | *ycf1* |  | (A)11 | 133746 | 133756 | *ndhG* |
|  | (A)10 | 143485 | 143494 | *ycf1* |  | (AATAT)3 | 135462 | 135476 | *ndhA* |
|  | (T)15 | 144254 | 144268 | *ycf1* |  | (T)10 | 135640 | 135649 | *ndhA* |
| ***G. japonica*** | (A)14 | 9189 | 9202 | *psbI* | ***G. sinensis*** | (A)12 | 9342 | 9353 | *psbI* |
|  | (T)10 | 28720 | 28729 | *rpoB* |  | (T)10 | 28798 | 28807 | *rpoB* |
|  | (A)10 | 74326 | 74335 | *rps18* |  | (T)10 | 60696 | 60705 | *atpB* |
|  | (T)14 | 117920 | 117933 | *ndhF* |  | (A)10 | 75257 | 75266 | *rps18* |
|  | (T)12 | 132431 | 132442 | *ycf1* |  | (T)15 | 118566 | 118580 | *ndhF* |
|  | (T)14 | 132667 | 132680 | *ycf1* |  | (T)12 | 132888 | 132899 | *ycf1* |
|  | (A)10 | 133679 | 133688 | *ycf1* |  | (T)14 | 133124 | 133137 | *ycf1* |
|  | (T)10 | 134203 | 134212 | *ycf1* |  | (T)11 | 133482 | 133492 | *ycf1* |
|  | (A)10 | 134807 | 134816 | *ycf1* |  | (A)10 | 134136 | 134145 | *ycf1* |
|  | (A)10 | 135387 | 135396 | *ycf1* |  | (A)10 | 135264 | 135273 | *ycf1* |
|  | (T)15 | 136156 | 136170 | *ycf1* |  | (A)10 | 135844 | 135853 | *ycf1* |
|  |  |  |  |  |  | (T)15 | 136613 | 136627 | *ycf1* |
